# Interactions between Triterpenes and a P-I Type Snake Venom Metalloproteinase: Molecular Simulations and Experiments

**DOI:** 10.3390/toxins10100397

**Published:** 2018-09-28

**Authors:** Lina María Preciado, Jaime Andrés Pereañez, Ettayapuram Ramaprasad Azhagiya Singam, Jeffrey Comer

**Affiliations:** 1 Programa de Ofidismo/Escorpionismo, Facultad de Ciencias Farmacéuticas y Alimentarias, Universidad de Antioquia UdeA, Medellín, Colombia; linampr@gmail.com (L.M.P.); jaime.pereanez@udea.edu.co (J.A.P.); 2 Institute of Computational Comparative Medicine, Department of Anatomy and Physiology, Kansas State University, Manhattan, KS 66506, USA; eazhagiy@vet.k-state.edu

**Keywords:** free energy calculation, adaptive biasing force, molecular dynamics simulation, snake venom metalloproteinase, triterpenes, enhanced sampling, explicit solvent

## Abstract

Small molecule inhibitors of snake venom metalloproteinases (SVMPs) could provide a means to rapidly halt the progression of local tissue damage following viperid snake envenomations. In this study, we examine the ability of candidate compounds based on a pentacyclic triterpene skeleton to inhibit SVMPs. We leverage molecular dynamics simulations to estimate the free energies of the candidate compounds for binding to BaP1, a P-I type SVMP, and compare these results with experimental assays of proteolytic activity inhibition in a homologous enzyme (Batx-I). Both simulation and experiment suggest that betulinic acid is the most active candidate, with the simulations predicting a standard binding free energy of $\Delta G^{\circ }=-11.0\pm 1.4$ kcal/mol. The simulations also reveal the atomic interactions that underlie binding between the triterpenic acids and BaP1, most notably the electrostatic interaction between carboxylate groups of the compounds and the zinc cofactor of BaP1. Together, our simulations and experiments suggest that occlusion of the S1${}^{\prime }$ subsite is essential for inhibition of proteolytic activity. While all active compounds make hydrophobic contacts in the S1${}^{\prime }$ site, β-boswellic acid, with its distinct carboxylate position, does not occlude the S1${}^{\prime }$ site in simulation and exhibits negligible activity in experiment.

## 1. Introduction

**Figure 1:**
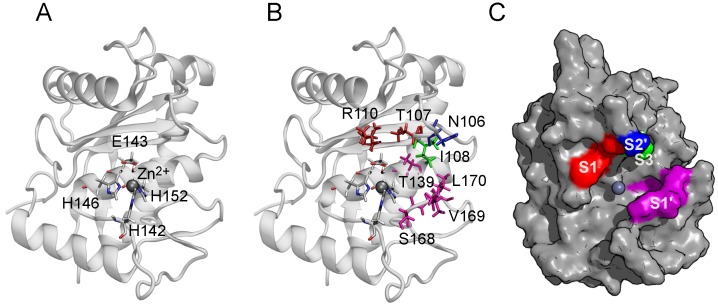
Structure of BaP1 and location of the catalytic zinc and the substrate binding subsites. (**A**) The catalytic zinc ion is coordinated tetrahedrally by the Nϵ2 atoms of three histidine residues (His142, His146, His 152) and the oxygen atom of the catalytic water molecule (Wat67), coordinated by the residue Glu143. The Zn${}^{2+}$ ion is shown as a gray sphere. The indicated amino acids are shown in a bond representation (H, white; C, gray; N, blue; O, red). (**B**) The side chains of amino acids belonging to the substrate binding subsites indicated by color: S1, red; S1${}^{\prime }$, magenta; S2${}^{\prime }$, blue; S3${}^{\prime }$, green. (**C**) Surface of BaP1 showing the substrate binding subsites with the color code previously described. Coordinates of BaP1 were obtained from the Protein Data Bank (PDB code 2W15).

Snake venom metalloproteinases (SVMPs) are zinc-dependent hydrolases that represent a major component in most viperid venoms and are classified into groups PI to PIII according to their domain organization [1]. Catalytic cleavage in SVMPs is mediated by a Zn${}^{2+}$ ion coordinated by three conserved histidine side chains, and a water molecule anchored to a glutamate residue. Consequently, the active site of these enzymes has the consensus sequence HEXXHXXGXXHD [2]. Following the common nomenclature for peptide/protease complexes, the metalloproteinase substrate binding site is divided into several subsites (regions on the enzyme surface that interact with individual amino acid residues on either side of the substrate cleavage site) [3]. The subsites located on the amino side of the cleavage region are labeled as S1, S2, S3 (unprimed subsites), while those on the carboxyl side are labeled as S1${}^{\prime }$, S2${}^{\prime }$, S3${}^{\prime }$ (primed subsites). The locations of these subsites in BaP1 are shown in Figure 1. These subsites contribute to metalloproteinase substrate selectivity and affinity [4].

SVMPs are responsible for the hemorrhagic activity induced by viperid venoms through hydrolysis of basement membrane proteins of capillary vessels and venules, including proteins such as type IV collagen, laminin, nidogen and perlecan [5,6,7]. In addition, there are secondary biological activities that are attributed to these enzymes, such as blistering, edema, myotoxicity, apoptosis and a prominent inflammatory reaction [8,9,10]. The only effective treatment for snakebite envenomations is the administration of antivenoms; however, the potency of these agents is sensitive to storage conditions [11,12] and they are limited in their ability to neutralize the toxins responsible for local tissue damage, due to its fast development [13]. Recently, snakebite envenomation was included on the World Health Organization list of Neglected Tropical Diseases, establishing renewed interest in promoting research and development of snakebite therapies [14]. The small molecule inhibitors against venom toxins show great promise, because they could be rapidly administered to arrest the progress of local tissue damage upon immediately after envenomation, complementing antivenom therapy. However, the safety of these inhibitors requires careful attention to inhibitor selectivity, because snake venom metalloproteinases possess catalytic sites similar to those in human matrix metalloproteinases, which are required for normal processes of extracellular matrix degradation and remodeling in the human body [15].

Different synthetic and natural compounds have demonstrated potential for neutralizing the proteolytic and pharmacological activities of SVMPs, including peptidomimetics Batimastat and Marimastat [16,17], zinc chelating agents such as EDTA, TPEN, DTPA or TTD [17,18], synthetic compounds, such as quinolinones [19] or thiosemicarbazones [20], and plant derived inhibitors, such as phenolic compounds [21,22], diterpenoids [23] and triterpenic acids [24]. In addition, computational approaches, such as molecular docking and molecular dynamics simulations have been used to study the interaction mechanisms of SVMP inhibitors and to identify new candidate compounds which were subsequently verified in experiments [20,25,26,27]. Molecular docking is commonly used to predict binding poses and to estimate binding affinity due to its low computational cost; however, molecular dynamics simulations coupled with approximate [28] or rigorous [29] free energy calculation techniques can provide more reliable results than the simple scoring functions used in docking.

Free energy methods follow two approaches: end-point and pathway. End-point methods estimate the binding free energy of the ligand and protein in unbound and bound states and calculate the binding free energy as the difference between these two states [30]. Examples of these methods include MM-GBSA, Ref. [31] and MM-PBSA. The latter technique has been applied to estimate binding free energies of various ligands to the P-I type metalloproteinase BaP1 from *Bothrops asper* venom [32,33]. Nevertheless, these end point methods are based on implicit solvent models and, therefore, neglect the granular nature of the solvent and lack a rigorous representation of hydrophobic effects [34]. In contrast, pathway methods rely on rigorous statistical mechanical techniques to sum free energy changes along a pathway connecting the initial and final state. Some examples include metadynamics [30], free energy perturbation [35,36] and adaptive biasing force (ABF) [37,38]. These methods are compatible with both implicit and explicit solvent models, with explicit solvent being generally more reliable and more computationally costly.

**Figure 2:**
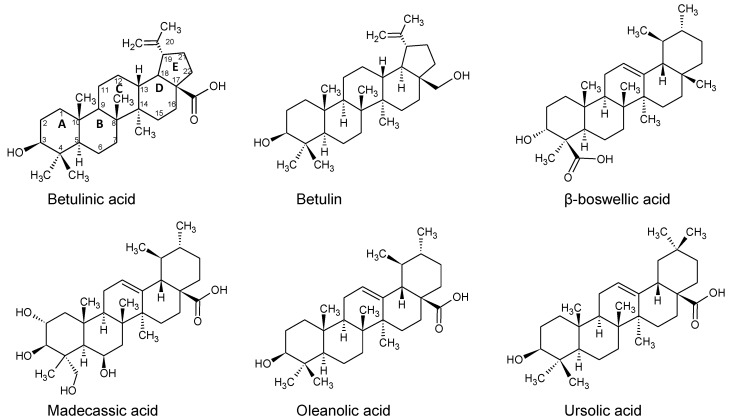
The chemical structures of the candidate compounds based on a pentacyclic triterpene skeleton. Carbon numbers and ring designations (A–E) are shown for betulinic acid.

While our experiments focus on Batx-I, a P-I type SVMP isolated from *Bothrops atrox* venom collected in Colombia, its complete sequence remains unknown and no structural information is available. BaP1 is a related enzyme from *Bothrops asper* with a published X-ray structure [39]. Patiño et al. [40] sequenced 15- and 9-residue fragments of Batx-I and found them to be identical to residues 7–30 of BaP1. Furthermore, they found that the proteolytic activity of Batx-I is comparable to that of BaP1—notably, in both cases, this activity is eliminated by EDTA, which chelates the catalytic Zn${}^{2+}$ ion. Thus, in this work, we use the ABF method and an approach similar to funnel metadynamics to compute the standard binding free energies and analyze the atomic interactions that give rise to the binding of betulinic, ursolic, oleanolic, β-boswellic and madecassic acids, as well as betulin (Figure 2), to the active site of the metalloproteinase BaP1. Some of these compounds were previously reported as inhibitors of the lethal, proteolytic and hemorrhagic activity of whole snake venoms and isolated metalloproteinases [24,41]. The computational results obtained through the ABF method are compared with experimental data of inhibition of the proteolytic activity of Batx-I.

## 2. Results and Discussion

First, we describe the determination of the inhibition constants and estimation of the standard binding free energies from the experiments. Then, in unbiased simulations, we unravel how the triterpenes bind to the metalloproteinase. The compounds having a carboxylate group at their C-17 position (betulinic, madecassic, oleanolic and ursolic acids) all show significant activity in experiment and highly favorable binding in simulation. The simulations demonstrate that binding of these compounds is driven by a strong electrostatic interaction between their carboxylate group and the catalytic Zn${}^{2+}$ ion of the metalloproteinase, which is further reinforced by hydrophobic interactions of the A, B and C rings. Betulin, which possesses a hydroxyl group at its C-17 position, exhibits negligible activity in experiment and a marginal binding free energy in simulation. All of the compounds having a carboxylate at the C-17 position bind with their A, B and C rings entering the S1${}^{\prime }$ subsite. For madecassic acid, occlusion of the S1${}^{\prime }$ is less complete than for the others, owing to hydroxyl groups on the A and B rings that reduce the hydrophobic interaction. This less complete occlusion may explain its reduced ability to inhibit proteolytic activity, as shown in the experiments. Likewise, β-boswellic acid, which has its carboxylate at the C-4 position, shows negligible activity in experiment. Although the simulations show favorable binding of β-boswellic acid to the metalloproteinase, the compound binds in a very different manner than the compounds having a carboxylate at the C-17 position, remaining far from the S1${}^{\prime }$ subsite. Hence, our experiments and simulations highlight the importance of the carboxylate–Zn${}^{2+}$ interaction and occlusion of the S1${}^{\prime }$ subsite for the inhibition of metalloproteinase proteolytic activity by triterpenic acids.

### 2.1. Inhibition of Batx-I Activity

$IC_{50}$ values for the inhibition of Batx-I proteolytic activity are displayed in Table 1. Betulinic, oleanolic and ursolic acids exhibited $IC_{50}$ values below 400 μM and were selected for further kinetic studies. Enzyme velocity as a function of the amount of substrate was used to fit the data with the Michaelis–Menten curve yielding the maximum velocity, $V_{\max}$, and Michaelis–Menten constant, $K_{M}$. Results showed that betulinic, oleanolic and ursolic acids increased the $K_{M}$ value without affecting the maximum velocity of the enzyme, acting as competitive inhibitors. Binding free energies derived from the ligand $IC_{50}$ values via the Cheng–Prusoff relation [42] are also shown in Table 1.

### 2.2. Binding Modes in Unbiased Simulations

**Figure 3:**
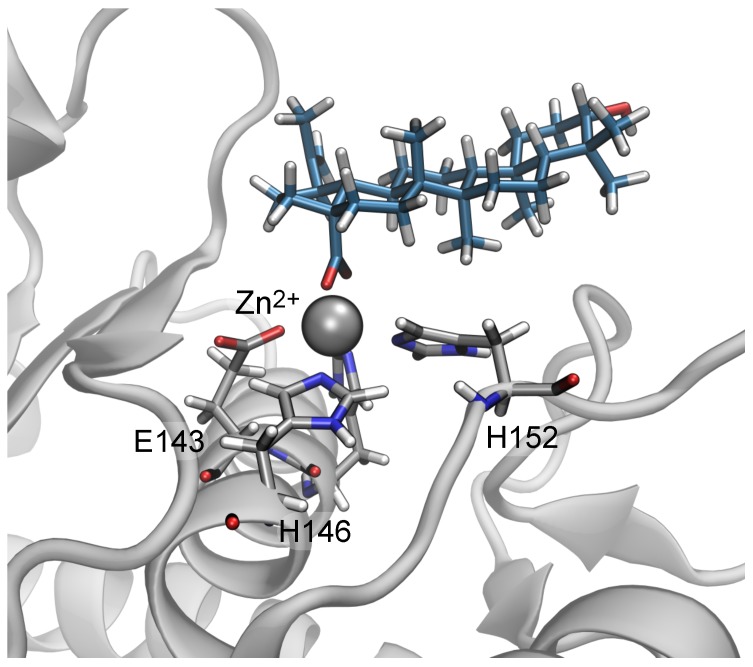
Snapshot from unbiased molecular dynamics simulations showing contact between the carboxylate group of betulinic acid and the catalytic Zn${}^{2+}$ ion of BaP1. Betulinic acid and indicated residues of BaP1 are are shown in a bonds representation with their carbon atoms colored cyan and gray, respectively. H, N, and O atoms are white, blue, and red. The Zn${}^{2+}$ ion is shown as a gray sphere. The protein secondary structure is represented in gray.

In unbiased simulations beginning from the poses predicted by molecular docking, the carboxylate group of the triterpenic acids rapidly made contact with the catalytic Zn${}^{2+}$ ion and the guanidinium group of the residue Arg110, despite the fact that these contacts were not present in the docking poses. A representative conformation of betulinic acid extracted from these simulations is shown in Figure 3. These simulations, which included explicit solvent, likely provide a more physically realistic representation than the “scoring function” used by docking programs. Furthermore, direct contact between the carboxylate and the Zn${}^{2+}$ ion seems plausible given the ionic interactions between the two, the importance of the Zn${}^{2+}$ ion in the catatlytic activity of the enzyme and experimental evidence showing that substitution of the carboxylate group with a hydroxyl group dramatically reduces the biological activity of the compounds (compare betulinic acid and betulin in Table 1). Hence, we hypothesized that the poses predicted by docking are unreliable and that the strong electrostatic interaction between the carboxylate group and Zn${}^{2+}$ ion revealed in explicit-solvent molecular dynamics simulation is a major contributor to the binding of the triterpenes to the metalloproteinase active site, followed by a minor contribution of a salt bridge with the guanidinium group of Arg110.

### 2.3. Binding of Triterpenes with Carboxylates at Position C-17

**Figure 4:**
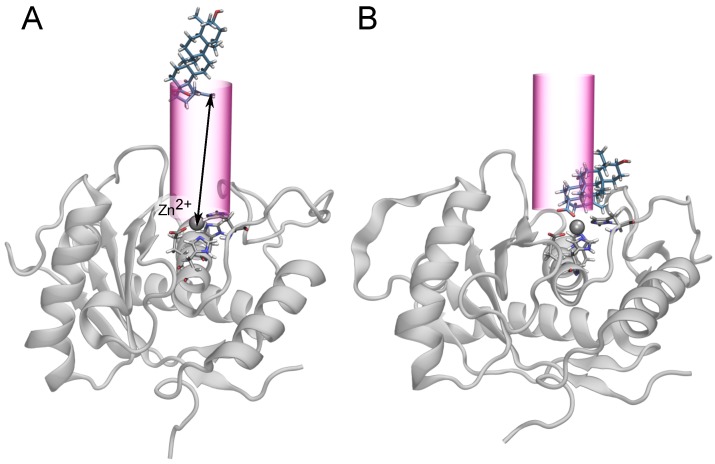
Schematic representation of the cylindrical restraint potential used in the free-energy calculations, similar to the funnel metadynamics protocol [30]. The carboxylate of the triterpenic acids (or C-17 hydroxyl of betulin) was subject to a restraining force when its distance from the cylindrical axis (centered on the Zn${}^{2+}$ ion) exceeded 4.5 Å. This distance was sufficiently large that the restraining force was never active when the carboxylate–Zn${}^{2+}$ distance was near the free energy minima (r<3.5 Å); therefore, it should not have influenced sampling of the bound state. The only exception was betulin, which possesses no carboxylate and exhibited marginal affinity for the active site. (**A**) An unbound state of belulinic acid and BaP1. The arrow represents the transition coordinate, *r*, defined as the distance between the carboxylate group and the Zn${}^{2+}$ ion. (**B**) A bound state of betulinic acid, with r=2.8 Å.

The unbiased simulations suggested a major role for the carboxylate–Zn${}^{2+}$ interaction in the binding of the triterpenes to BaP1. For this reason, we chose the distance between these two groups, *r*, as the transition coordinate for the free energy calculations. As described below, we also considered binding involving the guanidinium group of Arg110, but found less favorable free energies. Similar to funnel metadynamics [30], we applied a cylindrical potential so that large values of *r* correspond to the unbound state as illustrated in Figure 4. The influence of the restraining cylinder is accounted for in the calculation of the standard binding free energy (see Equation (2)). The simulations for each compound totaled 10–20 μs.

**Figure 5:**
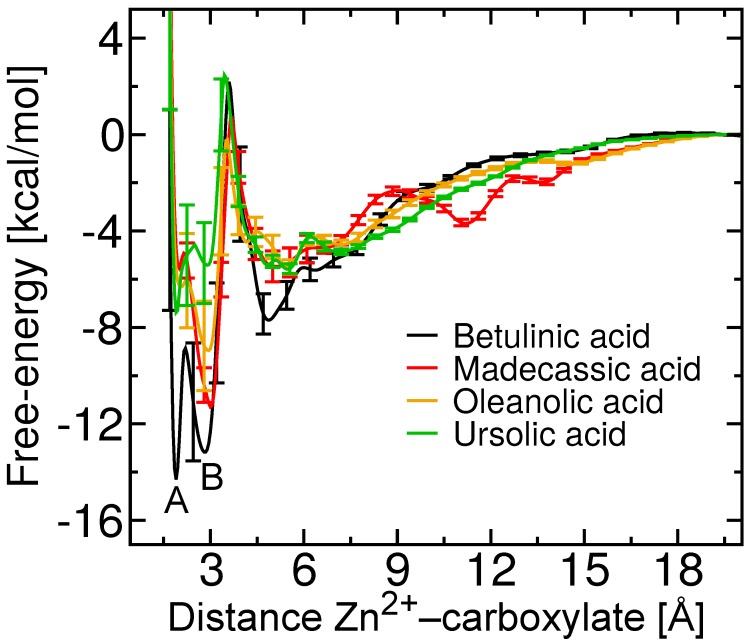
Potentials of mean force (Gibbs free-energy) as a function of distance from center of mass of the carboxylate group to the Zn${}^{2+}$ ion. The two different free energy minima for betulinic acid are marked as A and B.

The free energies as a function of the distance between the carboxylate group and the Zn${}^{2+}$ ion for betulinic, madecassic, oleanolic and ursolic acid, compounds with a carboxylate group at C-17, are shown in Figure 5. At long distances between the two groups, the free energy reaches a plateau, which implies that the interaction between BaP1 and the ligand is negligible at these distances. Thus, the free energy at r>19 Å is considered to be zero by convention. Decreasing distance between the carboxylate and Zn${}^{2+}$ ion is associated with favorable free energies, culminating in two distinct free energy minima. The relation of these two minima to the binding configuration is explored in the next section.

Following Limongelli et al. [30], we integrated the PMFs shown in Figure 5 to estimate the standard free energies for all triterpenes considered here, which are compared to the experimental estimates in Table 1. We do not expect quantitative agreement between the $\Delta G^{\circ }$ values derived from experiment and simulation, due to the fact that the experimental $\Delta G^{\circ }$ is determined indirectly from the enzyme kinetics. As described further below, the simulations suggest a break down of the assumptions of the Cheng–Prusoff equation (Equation (1)) used to obtain the experimental values, namely the existence of a single site of binding for the competitive inhibitor. Remaining discrepancies may be due to inherent differences between the proteins (Batx-I and Ba1P) considered in the experiments and simulations, imperfections in the computational representation of the atomic interactions or inadequate sampling in the ABF calculations. Experimental determination of the free energy of binding by direct techniques such as isothermal titration calorimetry might be compared more reliably with the simulation results, [43] although the relevance to the biological activity would be lost. Despite these limitations, both the simulations and experiments agree that betulinic acid possesses the greatest affinity for BaP1 of the compounds considered here. Furthermore, all compounds with a carboxylate at position C-17 (betulinic, madecassic, oleanolic and ursolic acid) show inhibitory activity in experiment and considerably favorable binding free energies in simulation. The apparent disagreement in Table 1 for β-boswellic acid appears to be due to its binding in a distinct configuration as explained in the section “Binding of Betulin and β-Boswellic Acid”.

When the carboxylate group of betulinic, madecassic, oleanolic and ursolic acids made contact with the Zn${}^{2+}$ ion, we observed displacement of the catalytic water molecule and its replacement by the carboxylate group of the amino acid Glu143. The loss of this water molecule likely contributes to the inhibitory activity of these C-17 carboxylate triterpenes. Displacement of the zinc-bound water molecule in the active site by other inhibitors of matrix metalloproteinases has been inferred in previous work [44].

**Figure 6:**
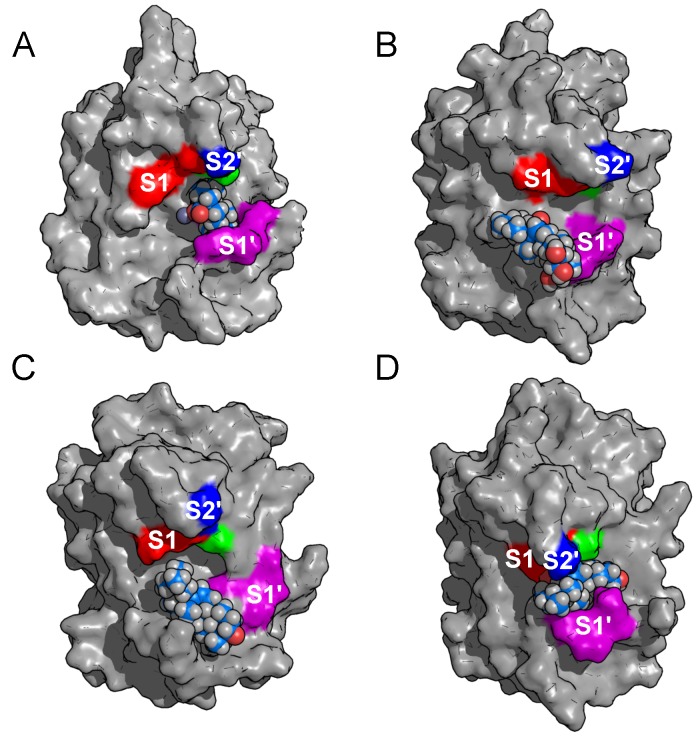
Conformations of triterpenes having a carboxylate group at position C-17 bound to BaP1. All these triterpenes partially occlude subsite S1${}^{\prime }$. (**A**) Betulinic acid. (**B**) Madecassic acid. (**C**) Oleanolic acid. (**D**) Ursolic acid. Subsites are colored and labeled as in Figure 1.

In addition, the orientations of triterpenes with carboxylate group at position C-17 bound to the metalloproteinase are shown in Figure 6, demonstrating that these compounds establish hydrophobic contacts between their A and B rings with amino acids belonging to the substrate binding subsite S1${}^{\prime }$. Nevertheless, betulinic acid is the only compound able to enter deeply into subsite S1${}^{\prime }$ (Figure 6A), which might explain its particularly high affinity for BaP1. On the other hand, as shown in Figure 6B madecassic acid occludes the S1${}^{\prime }$ binding subsite only partially, owing to substitutions of hydroxyl groups on A and B rings that reduce the hydrophobic interaction and, consequently, its experimental inhibitory activity.

Previously, Paes et al. [45] determined amino acid preferences for binding to S1${}^{\prime }$–S3${}^{\prime }$ of several snake venom metalloproteinases, including BaP1. All these SVMPs showed preferences for large, hydrophobic aliphatic residues occupying subsites S1${}^{\prime }$, S2${}^{\prime }$ and S3${}^{\prime }$. In addition, for matrix metalloproteinase inhibitors, it has been established that inhibitor specificity depends on hydrophobic extensions projected into the hydrophobic $S1^{\prime }$ pocket [46]. These observations for matrix metalloproteinases and SVMPs, enzymes that share high structural homology [47], are consistent with the hydrophobic contacts of betulinic acid and explain its greatest inhibitory activity. Thus, betulinic acid is a promising lead compound for the development of inhibitors of local tissue damage induced by snake venom, since all the SVMPs share highly similar zinc binding environments [10] and their amino acid preferences occupying S${}^{\prime }$ subsites are similar [45].

### 2.4. Two Binding Modes of Carboxylate–Zn${}^{2+}$

**Figure 7:**
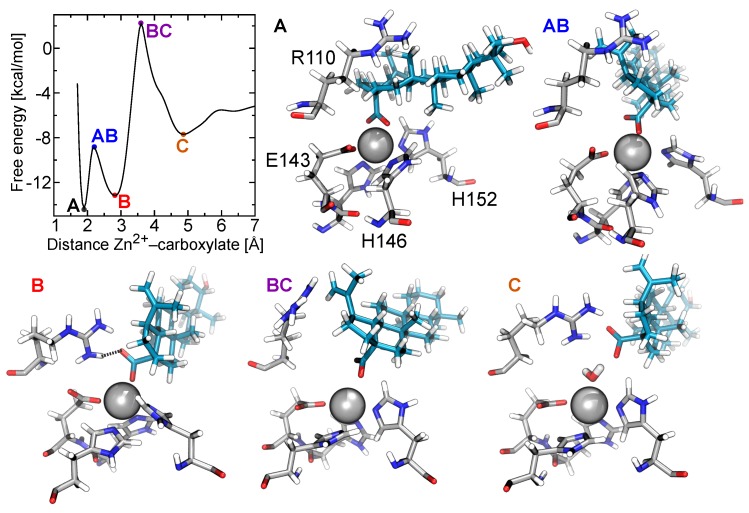
Representive configurations of betulinic acid and the metalloproteinase BaP1 corresponding to extreme points in the free energy plot. (**A**) Conformation of betulinic acid bound to BaP1 associated with the global free-energy minimum A, where both carboxylate oxygens make contact with the Zn${}^{2+}$ ion. (**AB**) Local free-energy maximum AB, where only one carboxylate oxygen is in contact with the Zn${}^{2+}$ ion, but there is insufficient space for the second oxygen to engage in other strong interactions. (**B**) Conformation associated with free-energy minimum B, where one carboxylate oxygen makes contact with the Zn${}^{2+}$ ion, while the other is hydrogen bonded to the Arg110. (**BC**) Local free-energy maximum BC, where the carboxylate is no longer in contact with the Zn${}^{2+}$ ion, but there is insufficient space for solvent between the two. (**C**) Conformation associated with free-energy minimum C, where the carboxylate and Zn${}^{2+}$ ion are bridged by a water molecule.

**Figure 8:**
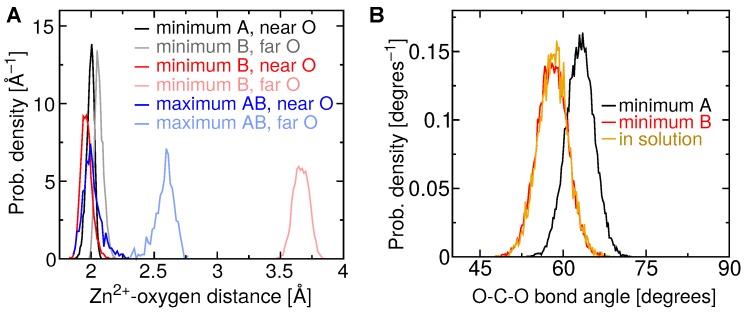
Geometry of the carboxylate of betulinic acid when bound to BaP1. (**A**) Histogram of the distance between each carboxylate oxygen atom and the Zn${}^{2+}$ ion for free-energy extrema described in Figure 7. (**B**) Histogram of the O–C–O bond angle adopted by the carboxylate group of betulinic acid near free-energy minima A and B, and in the aqueous phase.

The potentials of mean force in Figure 5 exhibit multiple free-energy minima. In all cases, two favorable minima lie at short distances between the carboxylate and Zn${}^{2+}$ ion, which we denote A and B. Betulinic acid clearly possesses two distinct minima, while free-energy minimum A is more weakly defined for the other triterpenic acids. Free-energy minimum A corresponds to a distance of r=1.90 Å between the center of mass of the carboxylate group and the Zn${}^{2+}$ ion. A representative structure for betulinic acid associated with this minimum is illustrated in Figure 7. The statistics of the geometry of this binding mode is shown in more detail in Figure 8A, where it can be seen that the two carboxylate oxygens are consistently found at a distance of 2.03±0.05 Å from the Zn${}^{2+}$ ion. Figure 8B indicates that the two carboxylate oxygen atoms straddle the Zn${}^{2+}$ ion, resulting in a mean O–C–O angle ($63^{\circ }$) larger than that for betulinic acid in solution. In this mode, no salt bridge forms with the guanidinium group of Arg110 (Figure S1A).

Free-energy minimum B occurs at a slightly larger distance between the center of mass of the carboxylate group and the Zn${}^{2+}$ ion, r=2.85 Å. As shown in Figure 7 and Figure 8A, only a single oxygen atom of the carboxylate makes contact with the Zn${}^{2+}$ ion in this mode, leading to an average O–C–O angle (58${}^{\circ }$) similar to that for betulinic acid in free solution. The second oxygen atom is relatively far from the Zn${}^{2+}$ ion, at a distance of about 3.6 Å, permitting it to form hydrogen bonds with water or Arg110. Some simulation frames associated with free-energy minimum B show this carboxylate–Arg110 contact, while others do not (see Figure S1B). In this free-energy minimum, hydrophobic contacts with Val169 and Leu170 are also established.

To better distinguish the binding free energy between the two modes for betulinic acid, we performed an additional set of ABF calculations, totaling 4 μs, on a small interval of the transition coordinate (1.7≤r≤3.0 Å), including little more than these two minima. The improved sampling in the neighborhood around the minima and the transition between them yielded sufficient precision to clearly determine which mode is the global free-energy minimum. Figure S5 verifies that minimum A is significantly more favorable than minimum B, with $\Delta G_{A}-\Delta G_{B}=-3.7\pm 0.4$ kcal/mol.

### 2.5. Free-Energy Barriers

The additional calculations on the interval 1.7≤r≤3.0 Å also refined the estimate of the sizable free-energy barrier between minima A and B for betulinic acid, namely 6.9±0.4 kcal/mol. This barrier is smaller but still present for the other triterpenic acids (see Figure 5). As exemplified in Figure 7, maximum AB, located at r=2.20 Å, is associated with a configuration in which only one of the carboxylate oxygen atoms makes contact with the Zn${}^{2+}$ ion, while the distance between the other carboxylate oxygen and the Zn${}^{2+}$ surface remains too tight to accommodate additional contacts. Figure 8A indicates that this second oxygen typically lies at a distance of <2.5 Å, which is too small for ingress of a water molecule. We find that the mean number of H-bonds donated by water to the carboxylate group is 2.56±0.08 for maximum AB, but rises to 2.82±0.09 at minimum B. Minimum A exhibits an even lower number of H-bonds between the carboxylate group and water (2.10±0.06), but the lack of solvation is presumably compensated by the strongly favorable carboxylate–Zn${}^{2+}$ straddling interaction.

All of the triterpenes considered here exhibit another free-energy maximum at r=3.6 Å, denoted BC, followed by a shallow free energy minimum near r=4.9 Å, denoted C (see Figure 5). As illustrated in Figure 7, maximum BC is caused by complete separation of the carboxylate group and Zn${}^{2+}$ ion while the intervening space remains too small to accommodate a water molecule. Further on, at minimum C, a water molecule is able to fit between the carboxylate and Zn${}^{2+}$ ion, making strong electrostatic interactions with both. Minimum C also appears to allow increased hydrophobic contact between the triterpenic acids and BaP1, compared to the more favorable minima A and B. States A, AB, and B have mean contact areas of 290–350 Å${}^{2}$ between BaP1 and betulinic acid, while states BC and C exhibit greater contact areas of 430 and 460 Å${}^{2}$.

### 2.6. The Role of Arg110

**Figure 9:**
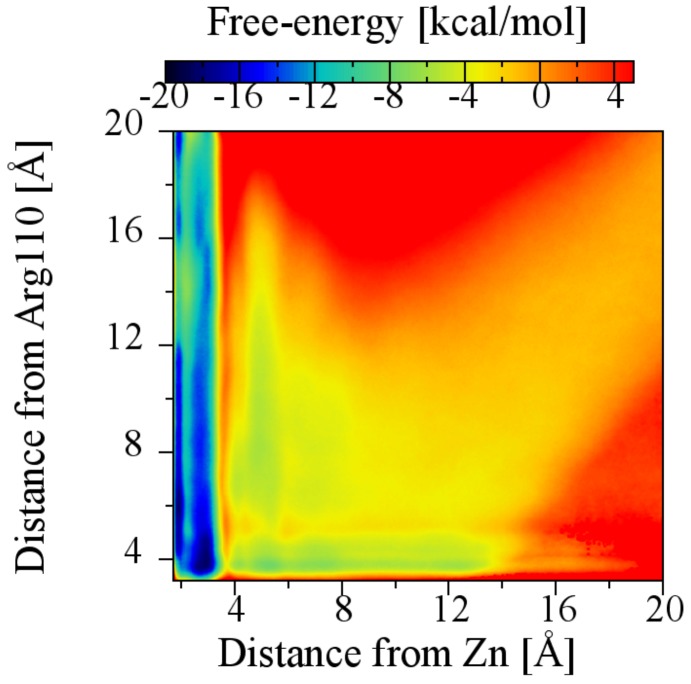
Plot of the two-dimensional potential of mean force (2D PMF) showing the dependence of the free energy landscape on the distance from the betulinic acid carboxylate group to the Zn${}^{2+}$ ion and to the carbon atom of the guanidinium group of the amino acid Arg110.

To determine a possible role for Arg110 in the affinity of triterpenes for BaP1, the ABF method was applied simultaneously to both the carboxylate–Zn${}^{2+}$ ion and the carboxylate–Arg110 transition coordinates (a two-dimensional calculation). In this case, a larger-radius (6.5 Å) cylindrical restraint was used to accommodate contact with Arg110 only. The two-dimensional (2D) potential of mean force, shown in Figure 9, suggests that the most important contribution to the free-energy minimum comes from the interaction with the Zn${}^{2+}$ ion, since the lowest free energy regions correspond to a distance of less than 3.7 Å between betulinic acid carboxylate group and the Zn${}^{2+}$ ion, with or without contact with residue Arg110. A distance of 3.8 Å between the carboxylate group and the guanidinium group, which is the minimum free energy when only the carboxylate–Arg110 interaction is considered (Figure S2), is associated with a higher value of free energy than the global minimum. Consistent with these result, alignments of the sequences of several viperid metalloproteinases (results not shown) reveal that Arg110 is not a conserved amino acid; thus, this interaction is likely not relevant for design of general SVMP inhibitors. We also do not know the identity of the corresponding residue in Batx-I—it may or may not be Arg.

In addition, ABF calculations with the transition coordinate defined as a function of the distance from the carboxylate group of the triterpenic acids (or the hydroxyl group of betulin) to the carbon of the guanidinium group of Arg110 were performed. In these simulations, the cylindrical restraint was defined to prevent direct contact between the triterpenes and the Zn${}^{2+}$ ion. The PMFs (Figure S2) for all the studied triterpenic acids one show a single free-energy minimum consistent with the formation of a salt bridge between the ligand carboxylate and Arg side chain, with an average distance of $r_{\mathrm{Arg}}=3.8$ Å. Free energy values for all the studied triterpenes with the Arg110 transition coordinate are displayed in Table S1.

Furthermore, we performed ABF calculations for betulinic acid applying different restraints to understand how the conformational changes in the protein can affect the binding free energy. Among other effects, the inclusion of these restraints prevented displacement of the Arg110 side chain and precluding its involvement in the binding process. As before, the transition coordinate was the distance between the carboxylate group of betulinic acid and the Zn${}^{2+}$ ion, with either the heavy atoms of the protein or its α-carbons restrained to their positions in the X-ray structure. The PMFs resulting from these calculations are shown in Figure S3. The standard free-energy values were ΔG=−10.1±0.1 and −12.6±2.1 kcal/mol, respectively, which are statistically consistent with the unrestrained value of ΔG=−11.0±1.4 kcal/mol. This result also further supports the assertion that the that the driving interaction for the binding of betulinic acid to the metalloproteinase BaP1 is the contact between the Zn${}^{2+}$ ion and the carboxylate group.

### 2.7. Thermodynamic Decomposition of Binding Free Energy

To better understand the thermodynamics of the binding of the triterpenic acids to BaP1, we decomposed the calculated free energy of betulinic acid into enthalpic (ΔH) and entropic (−TΔS) contributions [48,49]. The mean internal energies and pressure-volume works were calculated in the bound (for the free-energy minima A and B) and unbound states, and the resulting enthalpic and entropic components are listed in Table 2.

The driving force for binding associated with the free-energy minimum A comes from a favorable energetic contribution (ΔU=−12.2±0.3 kcal/mol), that reinforces a slightly favorable binding entropy (−TΔS=3.2±2.6 kcal/mol). This favorable energy is due to the strong electrostatic interaction between the two carboxylate oxygen atoms and the Zn${}^{2+}$ ion, and specific interactions with amino acids located at S1${}^{\prime }$ subsites. Simultaneously, these interactions impart conformational restrictions, yielding a less favorable entropy. On the other hand, the bound state associated with free-energy minimum B exhibits similar energetic (ΔU=−7.1±0.3 kcal/mol) and entropic (ΔU=−7.1±2.5 kcal/mol) contributions. The favorable energy comes from contacts between one carboxylate oxygen with the Zn${}^{2+}$ ion and the residue Arg110, while more favorable entropic contributions are due to the greater freedom of the compound relative to minimum A.

### 2.8. Binding of Betulin and β-Boswellic Acid

**Figure 10:**
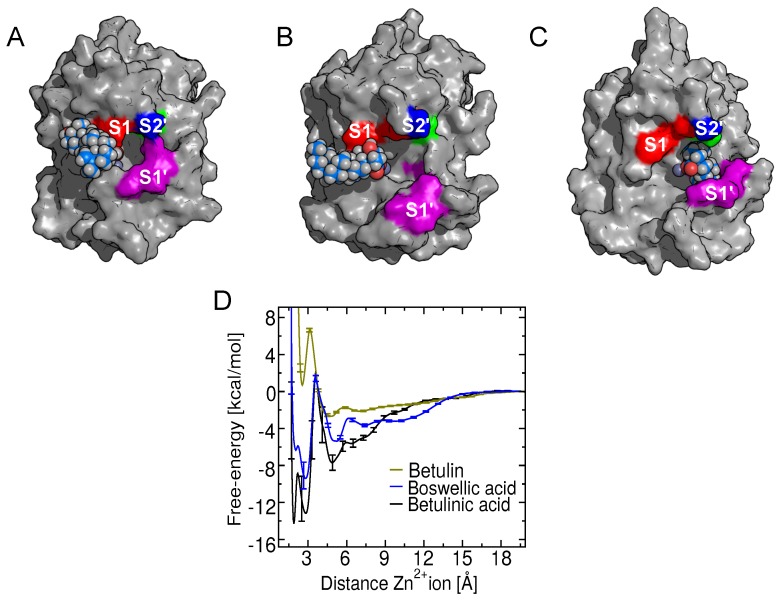
Comparison of the potentials of mean force (Gibbs free-energy) and the binding poses of betulin and β-boswellic acid with those of the most active compound betulinic acid. (**A**) Orientation of betulin associated with its free-energy minimum. The hydrophobic rings of betulin are interacting with the S1 subsite, without contact with the Zn${}^{2+}$ ion. (**B**) Orientation of β-boswellic acid associated with its free-energy minimum. (**C**) The orientation of bound betulinic acid is shown for reference. This compound fully occudes the S1${}^{\prime }$ subsite. (**D**) Free energy as a function of the carboxylate-Zn${}^{2+}$ ion distance for β-boswellic and betulinic acid, or hydroxyl-Zn${}^{2+}$ ion for betulin. The betulinic acid PMF is shown for comparison purposes.

Both experimental and computational results for betulin reveal a very low affinity for the metalloproteinase active site, with $\Delta G^{\circ }=-1.12\pm 0.01$ kcal/mol. In addition to its weak affinity, betulin was found to bind in an orientation distinct from the triterpenic acids having their carboxylate at the C-17 position, as shown Figure 10A. This distinct orientation may also contribute to its low activity.

Likewise, β-boswellic acid differs from the other triterpenic acids considered here in that its carboxylate group is attached to the C-4 atom of the triterpene skeleton rather than the C-17 atom. The PMF for this compound is presented in Figure 10C. The simulations for this compound demonstrate that it retains a strong interaction with the Zn${}^{2+}$ ion, yielding $\Delta G^{\circ }=-6.6$ kcal/mol. However, the experiments demonstrated that β-boswellic acid does not inhibit the metalloproteinase, even at high concentrations (mM range) (Table 1). The apparent discrepancy between the favorable binding predicted by simulation and the weak proteolytic activity observed in experiments is explained by the distinct configuration of β-boswellic acid in the BaP1 substrate binding site compared to (betulinic, madecassic, oleanolic and ursolic acids). When bound to BaP1 in its most favorable pose, the rings of β-boswellic acid are oriented toward the unprimed binding subsites of the enzyme, opposite the orientation adopted by triterpenes with their carboxylate at C-17. A comparison of the bound pose of β-boswellic in Figure 10B and that of betulinic acid in Figure 10C reveals stark differences in the contacts made by these compounds with amino acids of BaP1. While the C-17 carboxylate triterpenes all make contact with the S1${}^{\prime }$ subsite (Figure 6), β-boswellic acid lies several angstroms away, near the unprimed S1 subsite. The histograms in Figure S4 demonstrate that the configurations shown in Figure 6 and Figure 10 are representative and that β-boswellic acid only rarely contacts the S1${}^{\prime }$ subsite, while the other triterpenic acids spend most of their time there. This result, together with the lack of activity of β-boswellic acid measured in the experiments, suggests that occlusion of the S1${}^{\prime }$ subsite is necessary for inhibitory activity. Moreover, the trajectories for both β-boswellic acid and betulin revealed that the catalytic water is not displaced from the active site, which may also be linked with their poor inhibitory activity.

## 3. Conclusions

Explicit-solvent molecular dynamic simulations coupled with the adaptive biasing force (ABF) method allows estimation of the binding free energy and reveals the dominant atomic interactions underlying inhibition of snake venom metalloproteinases, which could be invaluable in the designing new inhibitors. We demonstrated that pentacyclic triterpenes having a carboxylate group at their C-17 position (betulinic, madecassic, oleanolic and ursolic acids) inhibit metalloproteinase proteolytic activity in experiment and exhibit favorable binding free energies and occlusion of the S1${}^{\prime }$ subsite in simulation. Our simulations showed that the binding of these compounds to BaP1 is mediated by a strong electrostatic interaction between the compounds’ carboxylate group and the catalytic Zn${}^{2+}$ ion, as well as hydrophobic interactions involving the A, B and C rings of the compounds and valine and leucine side chains located in the S1${}^{\prime }$ binding subsite. The importance of the carboxylate–Zn${}^{2+}$ interaction is highlighted by a comparison of betulinic acid and betulin, where replacement of betulinic acid’s carboxylate with a hydroxyl group leads to a dramatic loss of binding affinity and negligible proteolytic activity. Together, the simulations and experiments suggest that occlusion of the S1${}^{\prime }$ subsite is essential for the inhibitory activity. The most active compound, betulinic acid, also exhibited the deepest penetration of the S1${}^{\prime }$ subsite, while β-boswellic acid, a triterpenic acid having its carboxylate at position C-4, showed limited occlusion of the S1${}^{\prime }$ site and negligible inhibitory activity in experiments.

Despite qualitative consistency between the standard free energies derived from experiment and those calculated from simulation, quantitative agreement in these values remains elusive. A likely major contributor to the limited quantitative agreement seen in Table 1 is the calculation of the experimental $\Delta G^{\circ }$ from the enzyme kinetics, which is, by its nature, indirect. For example, a ligand may bind strongly to a protein, but away from the active site, yielding little or no effect on enzyme activity. This scenario appears to apply to β-boswellic acid: the simulations predict binding of similar strength to the active compounds, but with a very different orientation relative to the protein. Another contributor to the limited quantitative agreement might be the fact that the experiments and simulations analyze two similar, yet distinct, metalloproteinases. The experimentally derived values may also show sensitivity to the assay conditions [50], which may be difficult to reproduce precisely in simultion. Although our free-energy calculation for each compound involved four independent simulations and a total of 10–20 μs of simulated time, sampling the large number of degrees of freedom of the protein and compound is difficult and greater sampling may be needed to improve the accuracy of the calculations. Furthermore, the imperfections in the force field might contribute to some of the inaccuracy. The additive force field used in this work does not explicitly represent atomic polarizability, which is of particular for divalent ions, such as Zn${}^{2+}$; hence, the accuracy of the calculations might be improved by using a polarizable force field, for divalent ions, such as Zn${}^{2+}$ [51]. However, parameterization of general pharmaceutical compounds, such as the triterpenes considered here, remains a major undertaking for polarizable force-field frameworks.

The inhibition of the proteolytic activity of BaP1 shown here by betulinic, madecassic, oleanolic and ursolic acids, also implies a reduction of the hemorrhagic activity and tissue damage induced by snake venoms containing BaP1. Furthermore, the S1${}^{\prime }$ subsite, which was occupied by these triterpenic acids, differs substantially between SVMPs and matrix metalloproteinases found in humans, implying that they might exhibit selectivity for SVMPs over other metalloproteinases, reducing off-target effects. Hence, these triterpenic acids or engineered analogues could form the basis of a safe, rapidly accessible treatment for local damage against snake bites. Since snake venom metalloproteinases have a highly conserved catalytic domain with a consensus Zn${}^{2+}$-binding sequence, it seems likely that such compounds could be useful in preventing local tissue damage from snake bites by a wide range of viperid species. Design of novel terpene-based inhibitors could focus on maximizing affinity for SVMPs, while minimizing the affinity for human matrix metalloproteinases. Furthermore, the approach used here, based on molecular dynamics simulations and the ABF method, could also be valuable in the search of potent inhibitors of other snake venom toxins, for instance, phospholipases $A_{2}$.

## 4. Materials and Methods

### 4.1. Chemicals and Reagents

Betulinic acid, oleanolic acid, ursolic acid, β-boswellic acid, madecassic acid and betulin were purchased from Santa Cruz Biotechnology (Dallas, TX, USA). Other reagents used in this work were of the highest purity available from Sigma Aldrich (St. Louis, MO, USA) and Merck (Kenilworth, NJ, USA).

### 4.2. Purification of the Metalloproteinase Batx-I

Batx-I was isolated from a venom pool collected from adult specimens of *B. atrox*, which were gathered in the region of Meta in southeastern Colombia and maintained in captivity at the Universidad de Antioquia’s Serpentarium (Medellín, Colombia). Batx-I was purified via ion-exchange chromatography (CM-Sephadex C25) [40] and the toxin purity was judged by RP-HPLC and SDS-PAGE [52].

### 4.3. Inhibition of Proteolytic Activity

The inhibition of proteolytic activity was measured using the EnzCheck Gelatinase/Collagenase assay kit (Molecular Probes Inc., Eugene, OR, USA). All solutions and dilutions were prepared in assay-buffer (0.05 M Tris-HCl, 0.15 M NaCl, 5 mM CaCl${}_{2}$, 0.2 mM sodium azide and 1% DMSO by volume). The experiments were carried out using a 96-well plate, with a final volume of 200 μL. DQ-gelatin and the purified enzyme Batx-I were used at concentrations of 12.5 μg/mL and 21.4 μM. For all experiments, positive controls (the enzyme and assay buffer) and negative controls (assay buffer only) were included. The compounds were tested at concentrations ranging from 15 to 1500 μM. For the most active compounds, the $IC_{50}$ value was calculated from a dose–response plot. The fluorescence intensity was measured by a Synergy HT Multi-Mode Microplate Reader (BioTek Instruments, Inc., Winooski, VT, USA) for excitation at 485 nm and emission at 515 nm, at 1 min intervals for 60 min. Each reaction was performed in triplicate.

Analysis of the enzyme kinetics of Batx-I were carried out for betulinic, ursolic and oleanolic acids using a concentration of 800 μM for each compound. The substrate concentrations were in the range of 15.6 μmL to 2000 μg/mL in a 1/2 dilution series. For the calculation of substrate molarities, an approximate molecular weight of 100,000 g/mol was used [53].

The experimental standard free energy of binding was calculated with from the $IC_{50}$ value and kinetic parameters for betulinic, ursolic and oleanolic acids via the Cheng–Prusoff equation [42], which defines the theoretical relationship between the measured $IC_{50}$ for a competitive inhibitor using the substrate concentration (S) and the concentration of substrate at which enzyme activity is at half maximal ($K_{M}$):

(1) $$\Delta G^{\circ }=-kT\ln\left(\frac{IC_{50}}{1+S/K_{M}}\right)$$

### 4.4. Molecular Docking Methods

Molecular docking was carried out using Autodock Vina [54]. Although the sequence of Batx-I is not completely determined, the known portions are identical to residues 7–30 of BaP1 [40], and the two proteins have comparable biological activities. Therefore, the structure of the metalloproteinase BaP1 from *B. asper* venom (PDB code 2W15) [39] was used as a model toxin for the computational studies.

### 4.5. Molecular Dynamics Methods

The triterpenes were parameterized with the CHARMM General Force Field using the ParamChem web interface [55,56]. The negatively charged carboxylate state of the triterpenes dominates near neutral pH; hence, we constructed the computational models in this state. The metalloproteinase was represented in the simulations by the CHARMM36m force field for proteins [57,58] and constructed with the CHARMM-GUI server [59,60]. Conventional molecular dynamics simulations were performed with NAMD [61] and analyzed with VMD [62]. Lennard-Jones interactions were calculated with a 12 Å cutoff (standard for the CHARMM force field), smoothly truncated beginning at 10 Å. The pressure was maintained at 1.01325 bar using the Langevin piston method. The temperature was maintained at 310.15 K using a Langevin thermostat with a damping parameter of 1 ps${}^{-1}$. All simulations were performed with mass repartitioning of ligand and protein hydrogen atoms [63]. Electrostatic interactions were calculated using the particle-mesh Ewald method [64] with a grid spacing <1.2 Å. Water molecules were represented by the TIP3P model [65]. Sodium and chloride ions (0.15 M NaCl) were added to the aqueous phase. Additional ions were added to obtain charge neutrality.

### 4.6. Free Energy Calculations

The potentials of mean force were calculated by ABF [37,38] using the implementation provided in the Colvars module of NAMD [66]. To calculate the binding free energy of triterpenes to the metalloproteinase BaP1 active site, the radial transition coordinate *r* was defined as

$$r=\sqrt{{\left(X-X_{0}\right)}^{2}+{\left(Y-Y_{0}\right)}^{2}+{\left(Z-Z_{0}\right)}^{2}}$$

where (X,Y,Z) is the center of mass of the carboxylate group (O–C–O) of the triterpenic acids or the hydroxyl group of betulin and $\left(X_{0},Y_{0},Z_{0}\right)$ is the position of the Zn${}^{+2}$ ion or the carbon atom of the guanidinium group of Arg110. ABF was applied along the transition coordinate on the interval 1.7≤r≤20.0 Å using a bin size of 0.05 Å. The standard binding free energy was calculated by:

(2) $$\Delta G^{\circ }=-kT\ln\left(\pi R_{0}^{2}C_{0}\int dr\;\exp\left[-\beta \Delta G\left(r\right)\right]\right)$$

where $\beta =\frac{1}{k_{B}T}$ is the inverse thermal energy, $R_{0}$ is the radius of the restraint cylinder and $C_{0}$ is the standard concentration (1/1660.5389 Å${}^{3}$). In principal, Equation (2) should be corrected owing to the curvature of the radial coordinate; however, this correction is negligible at the unbound distance (*r* = 20 Å). A flat-bottomed harmonic restraint was applied to keep the carboxylate (or hydroxyl group for betulin) within a cylinder of radius $R_{0}=4.5$ Å, as shown in Figure 4. For each compound, four independent ABF runs were performed using the simulation conditions previously described. Each ABF run lasted 2.5–5.0 μs of simulated time, while the each of the four two-dimensional ABF runs reached 22 μs. The four independent calculations were appropriately combined (weighting the mean forces by the sample counts) to obtain the PMFs shown in Figure 5 and Figure 10D; hence, the curve for each compound represents 10–20 μs of simulation. Figure 9 comprises a total of 89 μs of simulation. Statistical uncertainties in the free energies were estimated from the differences between the four independent calculations.

To refine our calculation of the free energy difference between minimum A and B of betulinic acid, we performed four additional ABF calculations on the domain 1.8≤r≤3.0 Å, each having a length of 1 μs. As described by Comer et al. [38], we integrated the gradients and their associated uncertainties between minimum B r=2.85 Å and minimum A r=1.90 Å, to obtain the free energy difference and uncertainty estimate between the two minima. We also performed calculations following similar protocols to determine the interaction free energy between the guanidinium group of Arg110 of BaP1 and the carboxylate group of the triterpenic acids or the C-17 hydroxyl in the case of betulin, see Figure S2. For reference, we performed additional ABF calculations for BaP1 and betulinic acid with the heavy atoms or α-carbons of BaP1 restrained (Figure S3).

### 4.7. Determination of Binding Enthalpy and Entropy

To determine the contributions of enthalpy and entropy to the binding of BaP1 and betulinic acid [49,50], we performed equilibrium simulations in the free energy minima A and B, as well as the unbound state. The unbound state was maintained by applying a flat-bottomed harmonic potential active outside of the interval 28≤r≤32 Å, while similar restraints were applied to keep *r* near the value associated with the appropriate minimum (1.90 or 2.85 Å). In these simulations, restraining protein α carbons to accelerate convergence of the internal energy. As shown in Figure S3, the influence of these restraints on the free energies of the bound states was small. Each of the three simulations was run for 1.5 μs, with the potential energy of the entire system collected at 2 ps intervals. The average potential energies were computed after discarding the first 100 ns. The change in internal energy and volume for minimum A was calculated by $\Delta U_{A}=U_{A}-U_{\mathrm{unbound}}$ and $\Delta V_{A}=V_{A}-V_{\mathrm{unbound}}$, and similarly for minimum B. The entropic contribution to the binding free energy was calculated using ΔU and the minimum free energies: $-T\Delta S_{A}=\Delta G_{A}-\Delta U_{A}-p\Delta V_{A}$.

## Figures and Tables

**Table 1:** Experimentally determined inhibition constants and standard free energies compared to standard free energies calculated from simulation.

Terpene	${\mathrm{IC}}_{50}$ (μM)	$\Delta G^{\circ }$ Experimental (kcal/mol)	$\Delta G^{\circ }$ Theoretical (kcal/mol)
Betulinic acid	115±16	−4.5±0.1	−11.0±1.4
Ursolic acid	220±40	−3.5±0.1	−4.3±0.8
Oleanolic acid	360±70	−3.7±0.1	−6.1±0.6
Madecassic acid	540±80	ND	−5.8±0.5
Betulin	1470±130	ND	−1.12±0.01
β-Boswellic acid	ND	ND	−6.6±0.5

The $IC_{50}$ value of β-boswellic acid could not be determined because, at concentration of 2000 μM, 70% of Batx-I proteolytic activity was still observed. $\Delta G^{\circ }$ experimental was estimated from measurements of enzyme activity via the Cheng–Prusoff equation (Equation (1)). It should be noted that quantitative agreement between the experimental and theoretical $\Delta G^{\circ }$ values is not expected because the simulations suggest significant differences in the bound poses of the compounds, which are not captured by the Cheng–Prusoff equation, and, furthermore, the experiments were performed with Batx-I, for which no atomic structure is available; hence, the simulations used the homologous protein BaP1 [40].

**Table 2:** Decomposition of betulinic acid binding free energy into its enthalpic and entropic components.

Free-Energy Minimum	$\Delta G_{\min}$ (kcal/mol)	ΔU (kcal/mol)	pΔV (kcal/mol)	−TΔS (kcal/mol)
A	−15.4±2.1	−12.2±0.3	$\left(6.4\pm 1.5\right)\times 10^{-4}$	−3.2±2.6
B	−14.2±2.2	−7.1±0.3	$\left(9.0\pm 1.2\right)\times 10^{-4}$	−7.1±2.5

## Supplementary Materials

The following are available online at http://www.mdpi.com/2072-6651/10/10/397/s1. Table S1: Standard Gibbs free energies for binding to the guanidinium group of the residue Arg110. Figure S1: Distances between the betulinic acid carboxylate group and the Zn${}^{+2}$ ion or the carbon atom of the guanidinium group of residue Arg110 in the bound conformation. Figure S2: PMFs as a function of the distance from the center of mass of the carboxylate group of the triterpenic acids (or the hydroxyl group of betulin) to the central carbon atom of the guanidinium group of residue Arg110. Figure S3: PMFs as a function of distance from the center of mass of the carboxylate group of betulinic acid to the Zn${}^{2+}$ ion with restraints applied to the protein. Figure S4: Histograms of distances between the triterpenes and the S1${}^{\prime }$ subsite. Figure S5: Refined carboxylate–Zn${}^{2+}$ PMF between free energy minima A and B. Video S1: Conformation of betulinic acid bound to BaP1 showing contact of two carboxylate oxygens with the Zn${}^{2+}$ ion and hydrophobic contacts between its rings and amin oacids located at S1${}^{\prime }$ subsite (free-energy minimum A).

## Abbreviations

The following abbreviations are used in this manuscript:

ABF	Adaptive biasing force
COLVARS	Colective variables
EDTA	Ethylenediamine-tetraacetic acid
FES	Free energy surface
DTPA	Diethylene triamine pentaacetic acid
MM-PBSA	Molecular-mechanics Poisson–Boltzmann surface area
MM-GBSA	Molecular-mechanics Generalized Born surface area
PMF	Potential of mean force
RP-HPLC	Reverse phase-High-performance liquid chromatography
SDS-PAGE	Sodium dodecyl sulfate polyacrylamide gel electrophoresis
SVMPs	Snake venom metalloproteinases
TPEN	*N*, *N*, *N*’, *N*’-Tetrakis (2-pyridylmethyl) ethane-1,2-diamine
TTD	Tetraethylthiuram disulfide
