# Supplementary material for: Interactions between Triterpenes and a P-I Type Snake Venom Metalloproteinase: Molecular Simulations and Experiments
Source: Toxins (Basel). 2018 Sep 28;10(10):397. doi: 10.3390/toxins10100397 (PMC6215199; doi:10.3390/toxins10100397)
Supplement: Supplementary file 1 [file toxins-10-00397-s001.zip › toxins-354322-supple-final/toxins-35322-supple-final/supfile/toxins-353322-supple-final.pdf]

Supplementary Materials: Interactions between Triterpenes and a P-I Type Snake Venom Metalloproteinase: Molecular Simulations and Experiments

Lina María Preciado, Jaime Andrés Pereañez, Ettayapuram Ramaprasad Azhagiya Singam and Jeffrey Comer

Table S1. Standard Gibbs free energy derived from calculations where the transition coordinate was defined as the distance from the center of mass of the carboxylate group of the triterpenic acids (or the hydroxyl group of betulin) to the center of mass of the carbon atom of the guanidinium group of the residue Arg110.

Terpene	ΔG° theoretical (kcal/mol)
Betulinic acid	-4.4 ± 0.1
Ursolic acid	-4.5 ± 0.1
Oleanolic acid	-4.8 ± 0.1
Madecassic acid	-3.6 ± 0.1
Betulin	0.31 ± 0.01
Boswellic acid	-3.4 ± 0.1

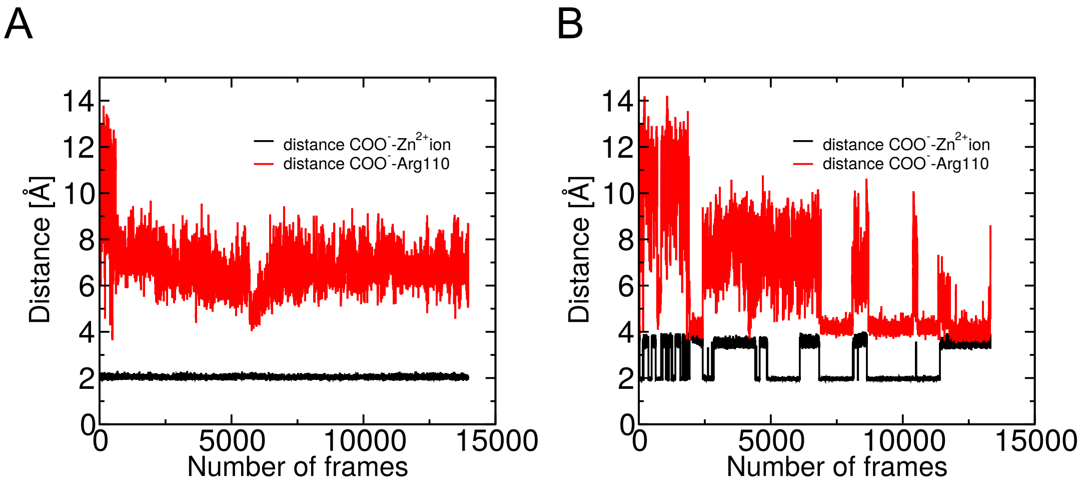

Figure S1. Distances between the carbon atom of the betulinic acid carboxylate group and the Zn^{+2} ion or the carbon atom of the guanidinium group of residue Arg110. (A) Distances in simulation frames associated with the global free-energy minimum A ($|r - r_A| < 0.1$ Å), for calculations with transition coordinate defined as the distance between the carboxylate group of betulinic acid and the Zn^{+2} ion. (B) Distances in simulation frames associated with the local free-energy minimum B.

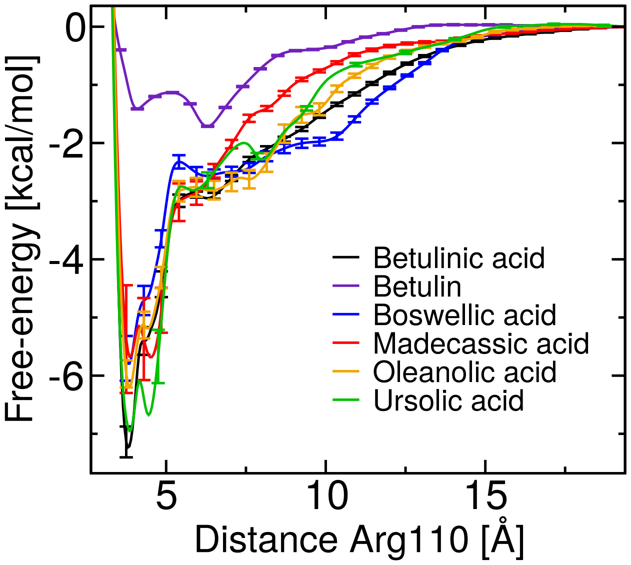

Figure S2. Potentials of mean force (Gibbs free-energy) as a function of the distance from the center of mass of the carboxylate group of the triterpenic acids (or the hydroxyl group of betulin) to the central carbon atom of the guanidinium group of residue Arg110. For betulin, the free energy minimum is associated with the formation of hydrogen bonds between its hydroxyl group at position C-17 and the guanidinium group.

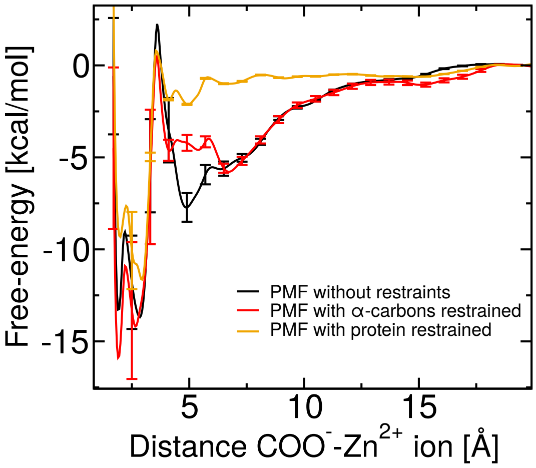

Figure S3. Potentials of mean force (Gibbs free-energy) as a function of distance from the center of mass of the carboxylate group of betulinic acid to the Zn^{2+} ion. These simulations were run under three different conditions: without restraints, α-carbons of the protein restrained and heavy (non hydrogen) atoms of the protein restrained.

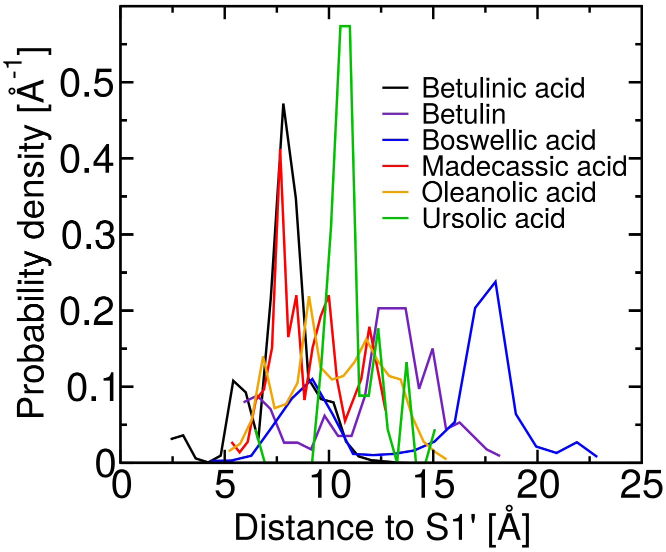

Figure S4. Histograms of distances between the center of mass of triterpenes and the center of mass of the S1′ subsite amino acids. These histograms were calculated from simulation frames associated with the global minimum free energy for the compound ($|r - r_{\rm min}| < 0.1$ Å).

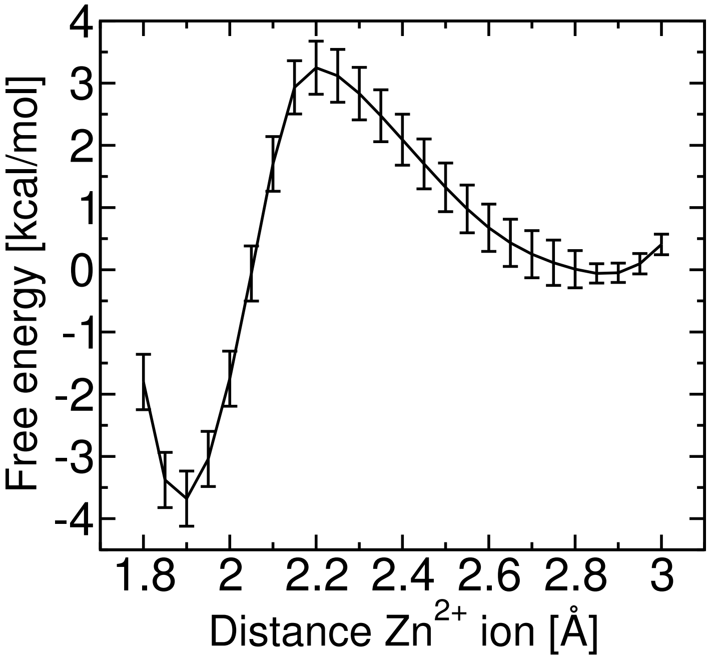

Figure S5. Potential of mean force as a function of distance from the center of mass of the carboxylate group of betulinic acid to the Zn^{2+} ion on the interval $1.7 \leq r \leq 3.0$ Å. Free energy minimum A, located at $r = 1.90$ Å, is clearly more favorable than free energy minimum B, located at $r = 2.85$ Å.
