# Supplementary figures and images for: Interactions between Triterpenes and a P-I Type Snake Venom Metalloproteinase: Molecular Simulations and Experiments
Source: Toxins (Basel). 2018 Sep 28;10(10):397. doi: 10.3390/toxins10100397 (PMC6215199; doi:10.3390/toxins10100397)

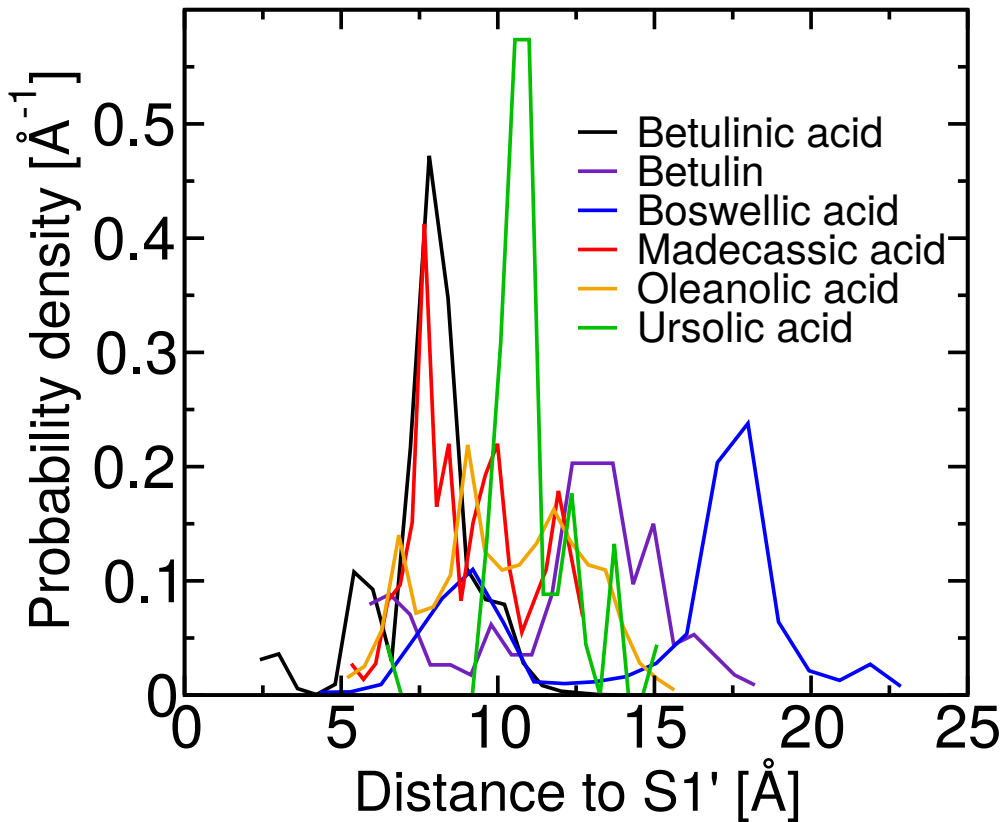

Supplement: Supplementary file 1 [file toxins-10-00397-s001.zip › toxins-354322-supple-final/toxins-35322-supple-final/supfile/distance_S1_correct.pdf]

**A**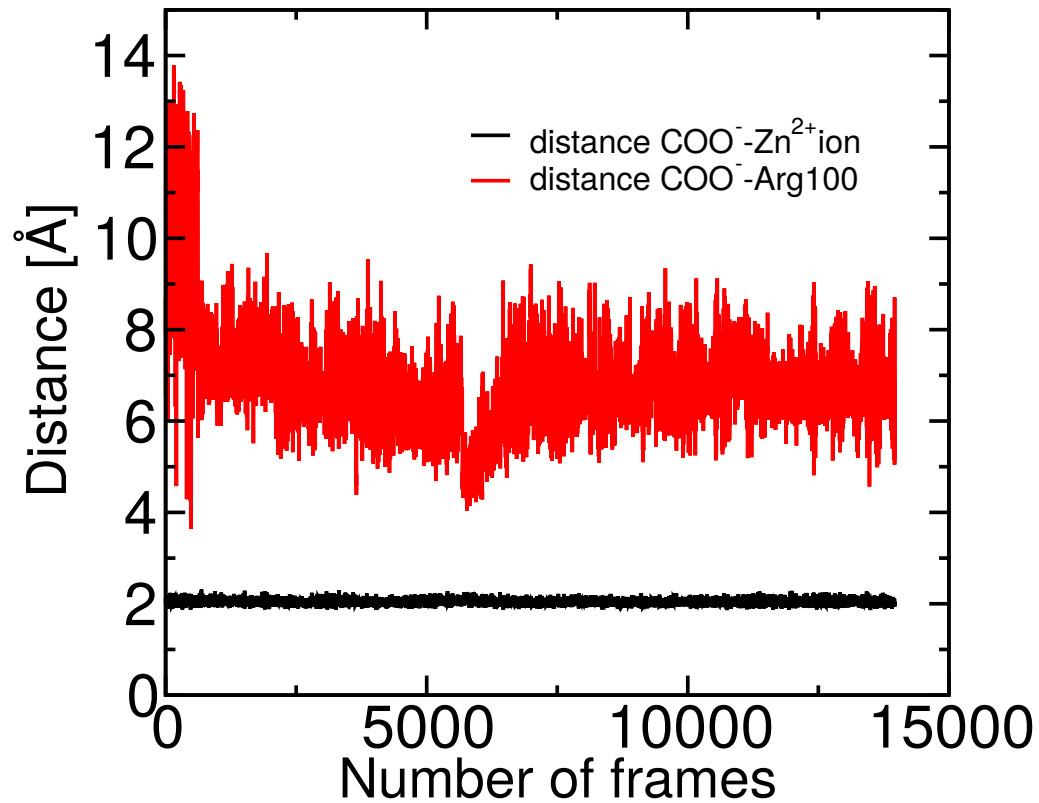**B**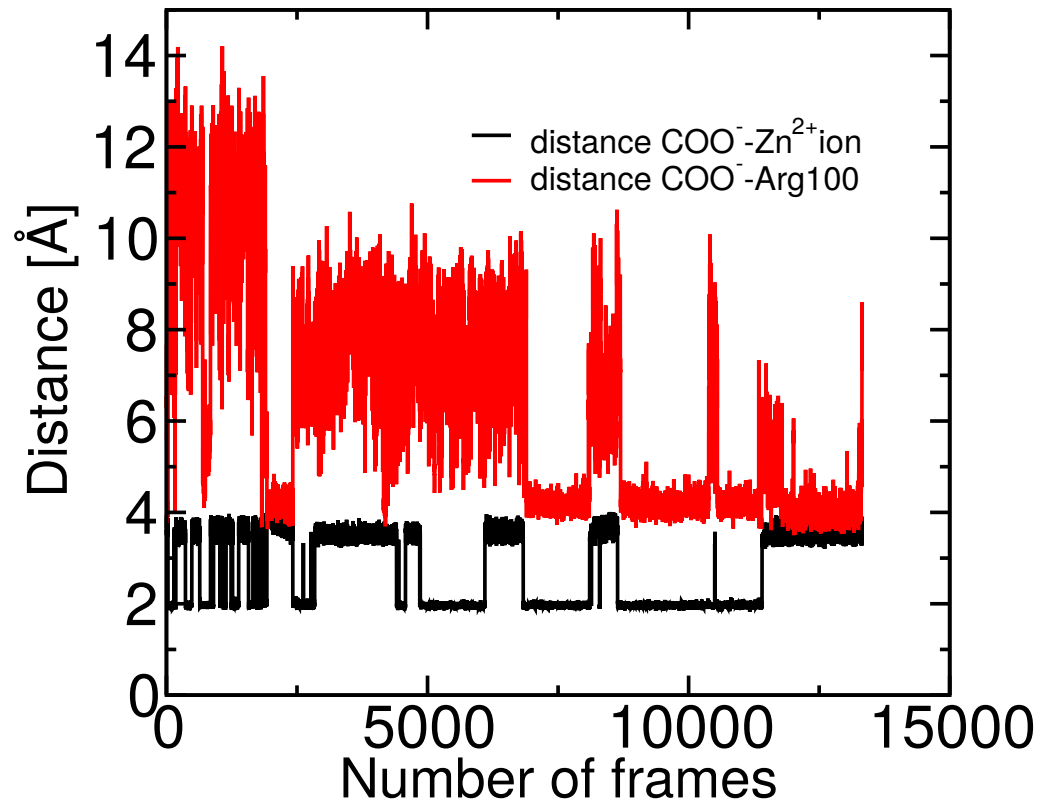

Supplement: Supplementary file 1 [file toxins-10-00397-s001.zip › toxins-354322-supple-final/toxins-35322-supple-final/supfile/Fig_S1.pdf]

**A**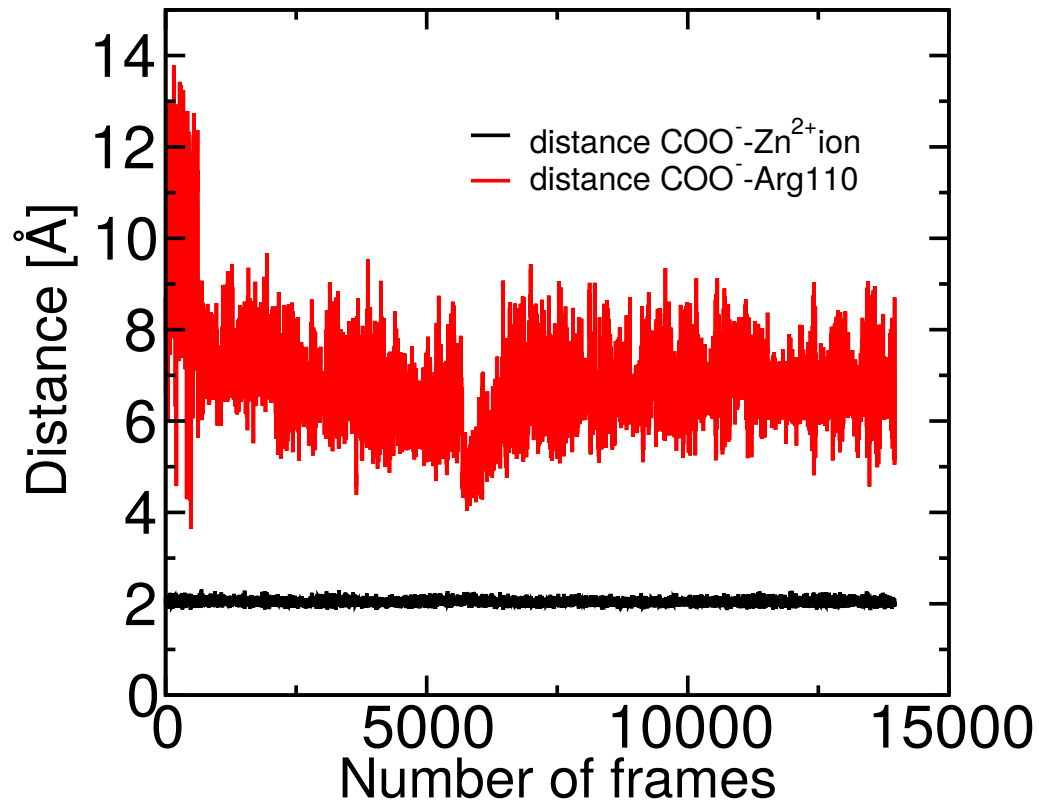**B**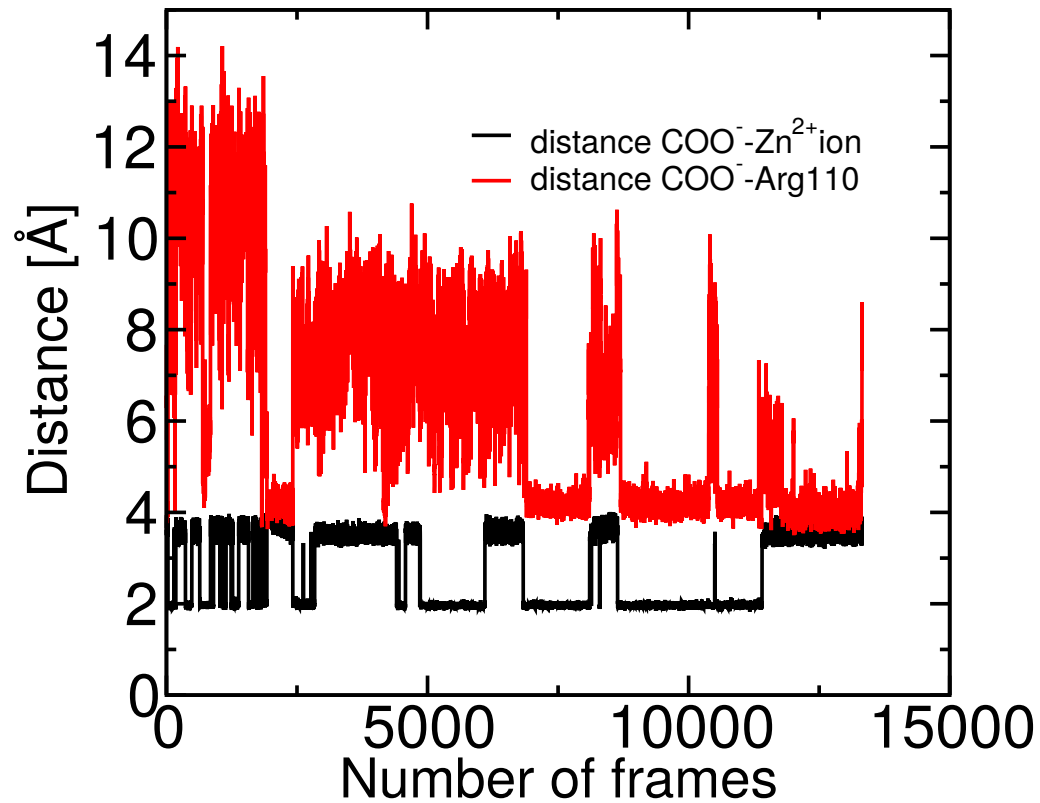

Supplement: Supplementary file 1 [file toxins-10-00397-s001.zip › toxins-354322-supple-final/toxins-35322-supple-final/supfile/Fig_S1_FINAL.pdf]

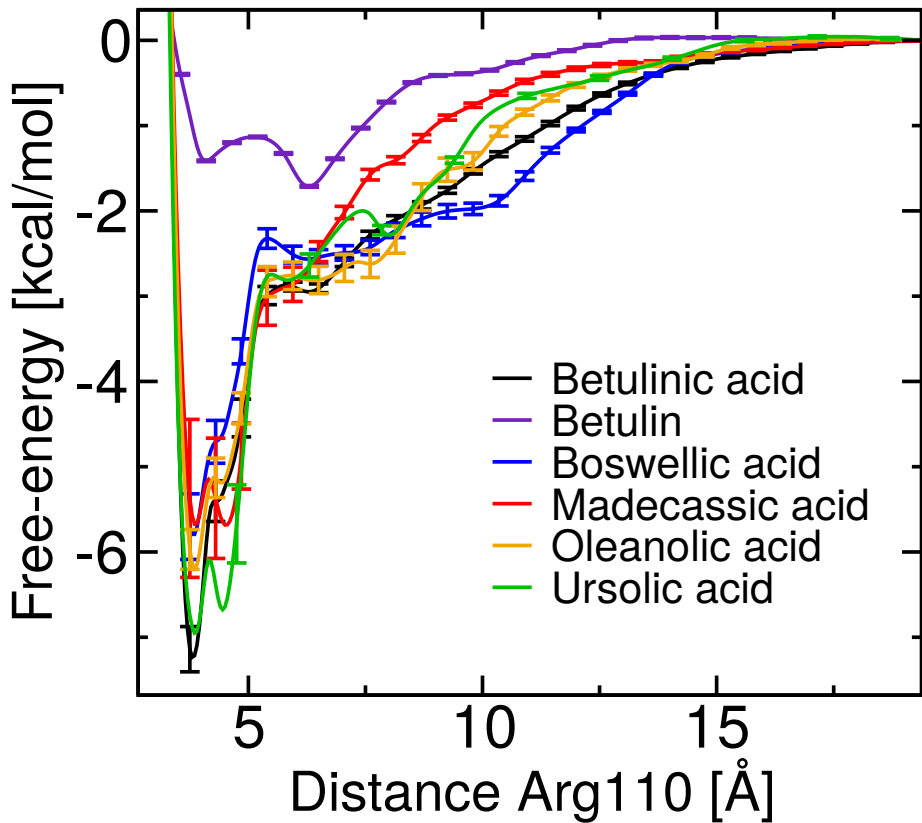

Supplement: Supplementary file 1 [file toxins-10-00397-s001.zip › toxins-354322-supple-final/toxins-35322-supple-final/supfile/Fig_S2.pdf]

Free-energy [kcal/mol]

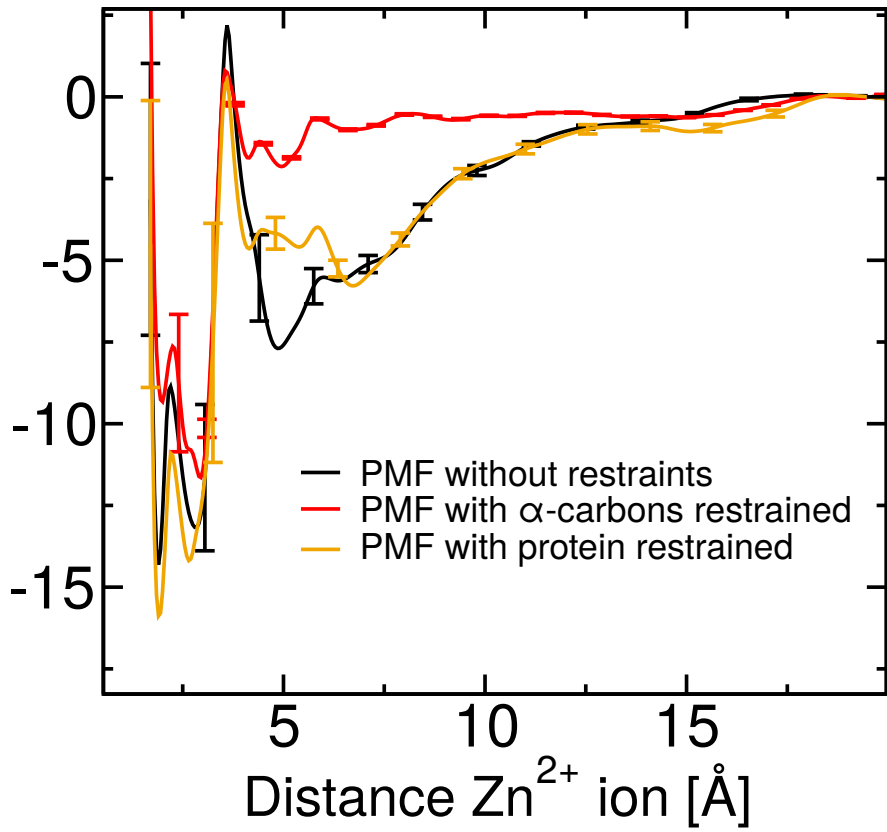

Supplement: Supplementary file 1 [file toxins-10-00397-s001.zip › toxins-354322-supple-final/toxins-35322-supple-final/supfile/Fig_S3.pdf]

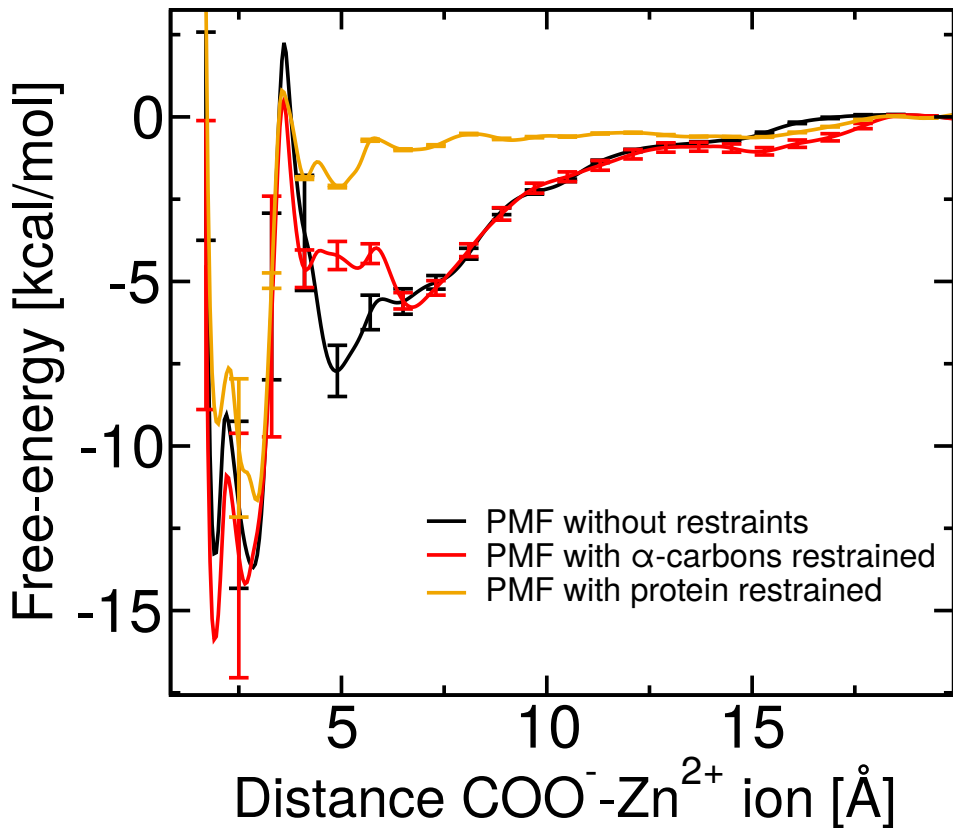

Supplement: Supplementary file 1 [file toxins-10-00397-s001.zip › toxins-354322-supple-final/toxins-35322-supple-final/supfile/Fig_S3_FINAL.pdf]

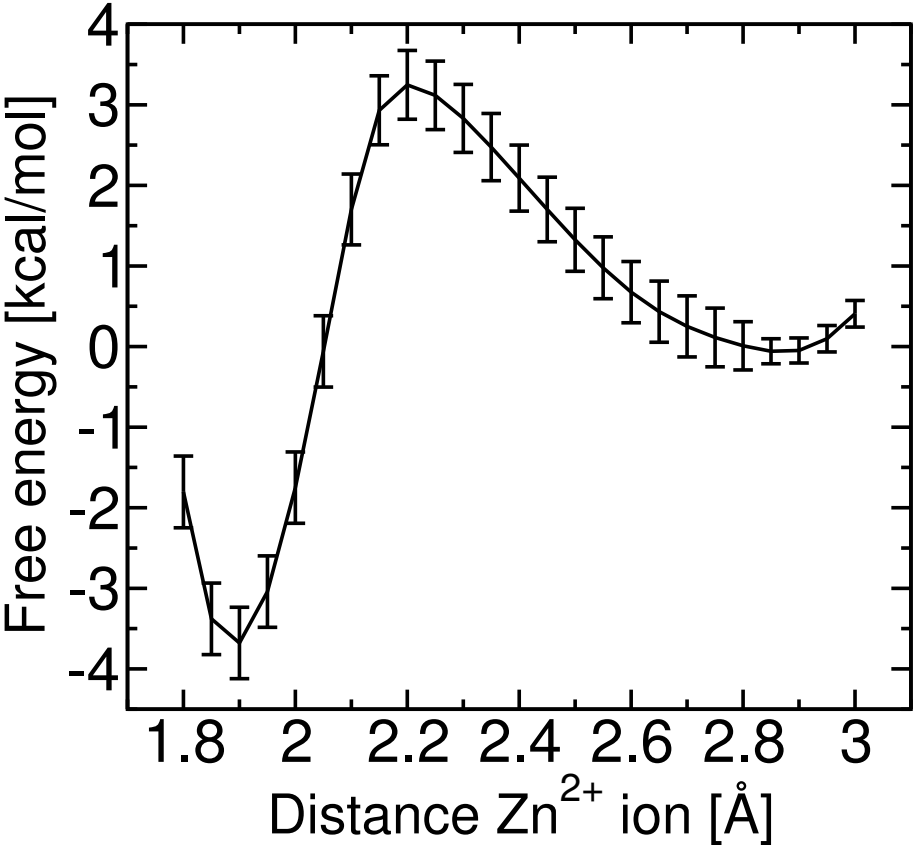

Supplement: Supplementary file 1 [file toxins-10-00397-s001.zip › toxins-354322-supple-final/toxins-35322-supple-final/supfile/plot_betu_window.pdf]
